# Toward Dynamically Adaptive Simulation: Multimodal Classification of User Expertise Using Wearable Devices

**DOI:** 10.3390/s19194270

**Published:** 2019-10-01

**Authors:** Kyle Ross, Pritam Sarkar, Dirk Rodenburg, Aaron Ruberto, Paul Hungler, Adam Szulewski, Daniel Howes, Ali Etemad

**Affiliations:** 1 Department of Electrical and Computer Engineering, Queen’s University, Kingston, ON K7L 3N6, Canada; 2 Faculty of Engineering and Applied Sciences, Queen’s University, Kingston, ON K7L 3N6, Canada; 3 Department of Emergency Medicine, Kingston Health Sciences Centre, Kingston, ON K7L 2V7, Canada; 4 Department of Chemical Engineering, Queen’s University, Kingston, ON K7L 3N6, Canada; 5 Department of Critical Care Medicine, Queen’s University, Kingston, ON K7L 2V7, Canada

**Keywords:** adaptive simulation, machine learning, wearable device, affective computing

## Abstract

Simulation-based training has been proven to be a highly effective pedagogical strategy. However, misalignment between the participant’s level of expertise and the difficulty of the simulation has been shown to have significant negative impact on learning outcomes. To ensure that learning outcomes are achieved, we propose a novel framework for adaptive simulation with the goal of identifying the level of expertise of the learner, and dynamically modulating the simulation complexity to match the learner’s capability. To facilitate the development of this framework, we investigate the classification of expertise using biological signals monitored through wearable sensors. Trauma simulations were developed in which electrocardiogram (ECG) and galvanic skin response (GSR) signals of both novice and expert trauma responders were collected. These signals were then utilized to classify the responders’ expertise, successive to feature extraction and selection, using a number of machine learning methods. The results show the feasibility of utilizing these bio-signals for multimodal expertise classification to be used in adaptive simulation applications.

## 1. Introduction

Simulations have been shown to be an important and effective tool for training that allow for experience to be gained through mimicking real world experiences in a risk-free interactive setting [1,2]. For instance, simulations are particularly useful for aiding trauma responders in becoming experts, or increasing their level of expertise, without any risk to patient safety [3,4,5,6]. By doing so, responders are able to learn how to make time-constrained life-and-death decisions, by applying knowledge learned in simulations to real world scenarios.

When designing simulations, it is essential for the learner’s initial level of expertise to be considered. If there is a discrepancy between their level of expertise, and that which the simulation is designed for, the learning outcomes will not be achieved, and there can even be negative implications on the learning objectives [7,8]. Such simulations can be designed to dynamically adapt to the participant’s expertise with respect to the task, enhancing the interactivity of the simulation and tailoring the learning experience to specific learners. This approach can allow for more complex scenarios to be introduced to novice learners gradually and based on learning progress, where eventually they can be exposed to simulations designed for challenging and cognitively demanding situations.

**Figure 1:**
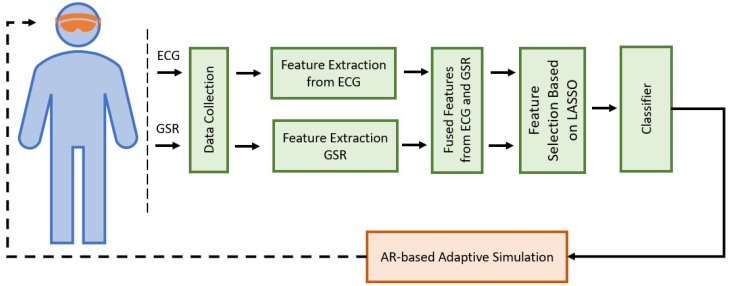
Proposed system architecture.

To ensure that the learning outcomes for simulations are better achieved, we propose an adaptive simulation paradigm in which the level of expertise of the participant is autonomously classified using their biometric signals with machine learning. This classification can then be used to adapt the simulation to the cognitive load of participants by altering the simulation difficulty. This classification can then be used to adapt the simulation to the cognitive load of participants by altering the simulation difficulty. The architecture for the proposed pipeline is shown in Figure 1. To support the proposed framework, the capability of wearable sensors to act as a meaningful input for machine learning classification of expertise and cognitive load must first be investigated. We chose to tackle this problem in the context of trauma medicine as a proof of concept. We specifically picked trauma medicine for investigating our proposed framework because it is possible to objectively distinguish between novice and expert trauma responders. Additionally, it has been shown that learner’s cognitive load, which itself is known to be directly impacted by the learner’s level of expertise, can significantly impact medical performance [9,10,11,12,13].

In this study, we investigate the applicability of electrocardiogram (ECG) and galvanic skin response (GSR) data for classification of expertise in the proposed adaptive simulation. This paper describes two trauma medicine simulations developed for the purpose of distinguishing between novice and expert trauma responders. ECG and GSR signals were collected using wearable sensors and used for expertise level analysis. In the following sections, we describe the materials and methods used in this study, followed by a detailed exploration of the ECG and GSR data for applications in classification of expertise. t-Distributed Stochastic Neighbor Embedding (t-SNE) is used to visually observe the separability of the feature space between experts and novices. Least absolute shrinkage and selection operator (LASSO) is then utilized to evaluate feature importance for use in expertise classification. Finally, classifiers are developed to differentiate expert and novice trauma responders using ECG and GSR features, both together and separately. These classifiers included support vector machine (SVM), decision tree (DT), random forest (RF), and K-nearest neighbour (KNN) models.

## 2. Related Work

In order to adapt a simulation to a user’s level of expertise, their expertise must first be detected and quantified. To the best of our knowledge, no research has been done on detecting learners’ level of expertise through biological signals. However, there has been found to be an inverse correlation between level of expertise and cognitive load [14]. Biometric measures, particularly heart rate variability (HRV) and Galvanic Skin Response (GSR), have been shown to be indicative of cognitive load [15]. Additionally, electroencephalogram (EEG) and electrooculogram (EOG) have been found to have a strong correlation with cognitive load [16]. These biological signals could also be indicative of level of expertise [17]. Machine learning classifiers using these bio-signals, could therefore, be used to facilitate dynamic classification of expertise for adaptive simulation.

Machine learning methods with which to identify cognitive load states have been investigated in [18,19]. In [18] ECG, electromyogram (EMG), respiration rate, GSR, and body temperature were utilized for the classification of overload, underload and normal cognitive load states. The performance of 3 different machine learning classifiers, namely KNN, naive Bayes, and random forest (RF), was then compared. The RF classifier was shown to perform the best, with a reported accuracy of 57.84%. In [19], a real time framework for classification of cognitive load was proposed. EEG and GSR signals were recorded from 10 participants with varying degrees of visual impairment as they navigated unfamiliar environments. An RF classifier was developed to classify low, medium, and high levels of cognitive load, using 5-fold cross-validation to achieve prediction rates of 83%–97%. These studies demonstrate the viability of real-time classification for cognitive load, and thus, most likely learner’s level of expertise.

When attempting to classify the level of expertise of a learner, it is important to take into account the stress that the individual is under. A person’s physiology can change when under high levels of stress [20] which may confound the assessment of cognitive load since it can elicit the same physiological responses [21]. As cognitive load has been found to be linked with expertise, the physiological changes from stress could also impact the ability of a classifier to elicit the participant’s level of expertise. However, some biological signals have been shown to be able to distinguish between the cognitive load of an individual and their level of stress. In [21], features extracted from peaks in GSR signals were able to identify high and low levels of cognitive load in both low stress and high stress conditions. This is important for our study as trauma responders are likely to face stressful situations in both simulation, and real world environments.

## 3. Materials and Methods

### 3.1. Simulation Design

**Figure 2:**
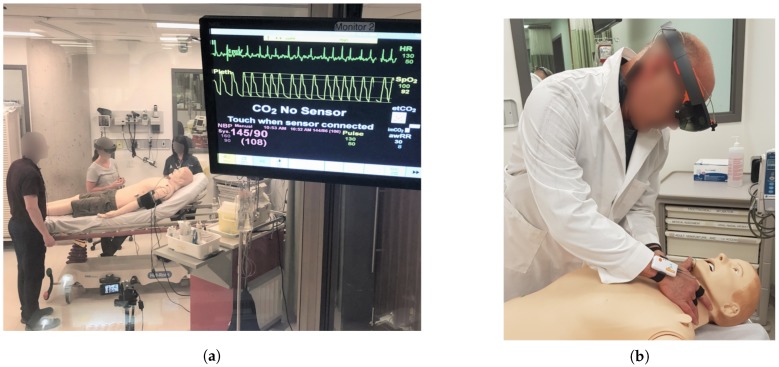
(**a**) Simulation environment with trauma team; (**b**) Instrumentation worn by participants.

As discussed in Section 1, the goal of this work is to utilize biological signals acquired through wearables to determine the users’ level of expertise. To this end, two separate trauma simulations were developed for the collection of biometric data from novice and expert trauma responders. A SimMan patient simulator (mannequin) [22] was used as the patient and was outfitted with artificial injuries. In one simulation, referred to as the Penetrating Trauma Simulation, the simulated patient had suffered a gunshot wound to the abdomen. In the second simulation, referred to as the Blunt Force Trauma Simulation, the simulated patient had been involved in an automobile roll-over resulting in blunt force trauma. Both simulations were designed to last 10 min. The vital signs of the simulated patient were controlled by a simulation technician throughout the simulation. First person videos of the simulations were recorded with a Microsoft HoloLens [23] worn by the participants. Shimmer3 wearable sensors were used to collect ECG and GSR data during the simulations [24]. The complete simulation environment, and instrumentation worn by the participants can be seen in Figure 2.

In the simulation, the participant played the role of a trauma team leader, directing the trauma team on how to provide care for the patient. The trauma team consisted of 1 registered nurse and 2 residents, all of which were hired actors. The participant was given a brief description of the trauma scenario prior to entering the simulation room. The goal of the participant during the Penetrating Trauma Simulation was early initiation of Massive Transfusion Protocol for resuscitation and disposition for emergency surgery. The goal of the participant during the Blunt Force Trauma Simulation was early identification and intervention of left sided tension pneumothorax (collapsed lung), followed by appropriate consultation and disposition with neurosurgery for further neuro-imaging, as well as thoracic surgery for consideration of operative intervention of left sided chest wall injuries, flail chest (broken rib cage), and pneumothorax. Participants completed both simulations successively, in random order.

Distractors were introduced verbally by the registered nurse to increase the cognitive load of the participants. These distractors included the introduction of an ECG reading with sinus tachycardia (a form of elevated heart rate), a high white blood cell count, and Emergency Medical Services (EMS) calling with a patch alerting the participant that a 60 year-old male with witnessed cardiac arrest is 5 min away from the emergency room. All of the distractors were used for each participant. After each of these distractors was introduced, the participant was asked by the trauma team what they would like to do with the information. This was done in order to distinguish differences between how novices and experts deal with the new information.

#### 3.1.1. Protocol and Subjects

Ethics approval was secured from the Queen’s University Research Ethics Board (QREB). Participants were recruited from two categories: expert and novice. The expert participants had completed their specialty training in emergency medicine and were practicing independent emergency medicine physicians. Moreover, they have had experience managing traumas as attending physicians. The novice participants were Queen’s University medical students at the end of the 4th year of their medical studies. They had been rotated through multiple medical specialties (i.e., internal medicine, surgery, emergency medicine, etc.). The reason the novice group was selected from students with some background in trauma medicine, was so that they would have the knowledge necessary for being able to treat the patient successfully. A total of 10 participants were recruited, 5 experts (3 male, 2 female) and 5 novices (2 male, 3 female). The ages of the expert participants ranged from 31 to 44, while the novices ranged from 25 to 34. In addition to data collection during the simulation, baseline data were collected for 2 min prior to the start of the simulation in a quiet room, while participants were in a relaxed seated position.

#### 3.1.2. Sensors and Data

Shimmer3 ECG and GSR wearable devices [24] were used in this study, as seen in Figure 2, to collect data during the simulations. The sensors were small and lightweight, with the ECG sensor weighing 31 grams, while the GSR sensor weighs 28 grams. The Shimmer3 ECG sensor allows for ECG signals to be measured from four bipolar limb leads in addition to one chest lead. The signals obtained from the differential ECG channel between Left Arm (LA) and Right Arm (RA) limb leads were used in this study. The Shimmer3 GSR sensor collects one channel of GSR data by monitoring the conductivity between two reusable electrodes that can be attached to two fingers using velcro straps. The GSR sensor itself was clipped to a strap on the participant’s wrist, while the ECG sensor was clipped to a strap placed around the waist of the participant. The signals from both sensors were obtained over Bluetooth connection allowing for full mobility of the participants during the simulation. Both signals were collected at a sampling rate of 500 Hz.

### 3.2. Analysis and Classification

#### 3.2.1. Pre-Processing

**Figure 3:**
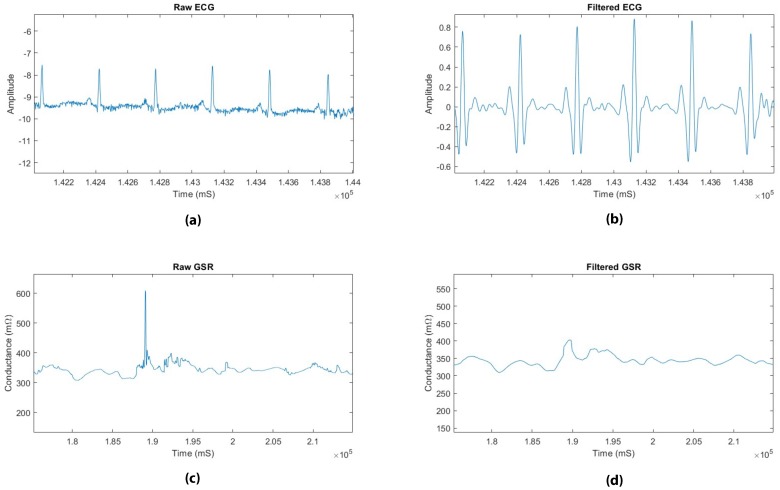
Examples of: (**a**) Raw electrocardiogram (ECG) signal; (**b**) Filtered ECG signal; (ECG) (**c**) Raw galvanic skin response (GSR) signal; (**d**) Filtered GSR signal.

The Pan-Tompkins (PT) algorithm was utilized to process the ECG signals and detect the QRS complexes [25,26]. In this algorithm, to reduce the influence of electromyogram (EMG) noise, powerline noise, baseline wander, and T-wave interference, a Butterworth bandpass filter was applied with a passband frequency of 5–15 Hz. The signal was then differentiated using a 5-point derivative transfer function to provide the QRS slope information. The absolute value of the signal was taken and a moving average filter was used to obtain the wave form features in addition to the R-peaks. Examples of the raw and filtered ECG signals are shown in Figure 3.

**Figure 4:**
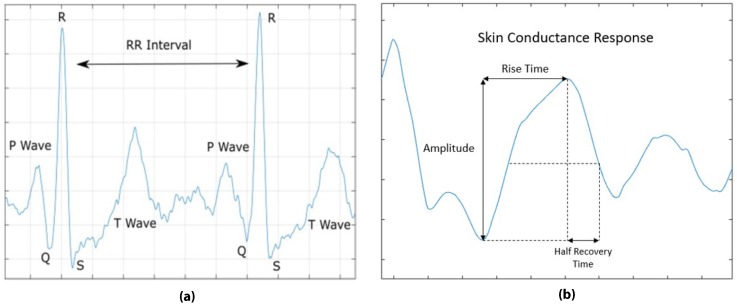
(**a**) Example of the interval between R peaks (RR interval) from ECG signal; (**b**) Example of Skin Conductance Response (SCR) from GSR signal.

A moving 150 ms window was employed to obtain features from the filtered signal by first detecting the R-peaks. To detect the R-peaks, two threshold values were selected to distinguish between the peaks and noise. If no peaks were detected in a time window of two seconds, a search-back technique was initiated to find the missed R-peaks. The threshold values were set iteratively based on the most recent detected signal and noise peaks. All features were obtained from the intervals between two R-peaks (RR interval). An example of an RR interval is shown in Figure 4.

The GSR signals were first filtered following the method used in [27] by first using a low-pass filter with a cutoff frequency of 1 Hz. Additionally, high-frequency artifacts were removed using a moving average filter with a filter size of 1000 samples. An example of the raw and filtered GSR signals is shown in Figure 3. Next, Skin Conductance Response (SCR) events were identified by detecting the local peaks in the GSR signal. An example of an SCR event is shown in Figure 4.

#### 3.2.2. Feature Extraction

Next, to identify important changes in the ECG signals, a large number of features are extracted from the data recorded during the simulation. To this end, we first segmented the data into 10-s windows with an overlap of 5 s. The window size was selected similar to [28] and successive to trial and error with the goal of maximizing classifier performance. Similar to [29,30], 11 time domain features and 8 frequency domain features were calculated. All features were extracted from both the baseline data and simulation data. The features from the simulation data were normalized with respect to the baseline data by dividing the values of the features by that of the corresponding baseline features. For time domain features, statistical features were extracted from the RR intervals of the ECG data. A Lomb periodogram [31] technique was used for power spectrum density (PSD) analysis to obtain frequency domain features. The features extracted from the ECG signals are summarized in Table 1.

The GSR signals from the simulation were segmented into 30-s windows with 10 s of overlap following [32] and subsequent to trial and error for maximizing the performance of our system, as well as ensuring that at least one SCR event is captured in the selected time window. Similar to [27], 8 time domain features, and 2 frequency domain features were extracted from the SCR events of both the baseline data and simulation data. In cases where multiple SCR events were found in a window, the mean, minimum, maximum, and standard deviation of each time domain feature was calculated and subsequently used. Additionally, the Skin Conductance Level (SCL) was determined by taking the average of the GSR signal in the time window. All of the features extracted from the GSR signals are summarized in Table 2. The baseline features were used to normalize the features extracted during the simulation by dividing those features by the corresponding baseline feature.

#### 3.2.3. Feature Space Exploration

The usefulness of the extracted ECG and GSR features for expertise classification was examined by reducing the high dimensionality of the respective feature spaces to 2 dimensions with *t*-SNE [33]. This allowed for the visualization of the feature space in order to evaluate the separability of the two classes. *t*-SNE was performed using 10,000 iterations with a perplexity of 30, and learning rate of 10. Each 10 s time window of ECG data was treated as a separate sample, while every 30-s time window of GSR data was treated as a sample. The samples were assigned a class according to the expertise of the participant.

#### 3.2.4. Feature Selection

To determine which of the features from the ECG, GSR, and multimodal feature sets are important for the classification of expertise, LASSO [34] was used. LASSO is a regression analysis method where the absolute size of the regression coefficients are penalized by reducing them. After the process, the features with non-zero coefficients are suitable for use in models [35]. The larger the magnitude of the coefficients, the greater the importance of that feature for differentiation of the two classes. In our study, LASSO was used to calculate the regression coefficients for each feature.

#### 3.2.5. Classification

Several classifiers were utilized to differentiate levels of expertise. The classifiers were trained separately with the important features from ECG, GSR, and multimodal feature sets. Four different supervised models were used to develop separate classifiers in order to compare the performance.

Support Vector Machine is a machine learning classification technique in which the features, or input vectors, are mapped onto a high dimensional feature space. The feature space is then divided using a hyperplane to separate the features of different classes such that the margin between features of different classes is maximized [36]. The mapping of the features onto a higher dimension feature space is determined by a kernel function selected for the SVM. Multiple kernels, namely linear, quadratic, and radial basis function (RBF), were experimented with, to compare the performance. The best performance was obtained using a second degree polynomial kernel.

Decision Tree classifier uses several decision functions successively to classify an unknown sample. The decision functions take the sample from a root node, through interior nodes to a terminal node that represents its classification [37]. Random Forest classifier is an ensemble of decision tree classifiers that each generate a classification decision. The most popular class from the decision trees is returned as the overall classification outcome [38]. The number of trees in the forest were changed iteratively to obtain the highest accuracy. It was found that a random forest of 100 trees achieved the best results.

K-Nearest Neighbours classifier determines the class of a sample by determining the most popular class from the nearest set of training samples [39]. The variable *k* denotes the number of neighbours in the training set used for classifying an input test sample. In our experiments, *k* = 5 provided the best results when used with a uniform Euclidean distance function.

#### 3.2.6. Evaluation

To evaluate the performance of the model, both accuracy (Acc.) and F1 score were calculated using leave-one-subject-out (LOSO) validation scheme. True Positive (*TP*) and True Negative (*TN*) measures were the number of correctly classified expert and novice participants, while False Positive (*FP*) and False Negative (*FN*) were defined as the number of incorrect classifications. Accuracy is defined as the percentage of the total number of correctly classified participants to the total number of participants, expressed in Equation (1).

(1) $$Accuracy=\frac{TP+TN}{TP+FP+TN+FN}$$

The F1 score is a metric that combines precision and recall. Precision is the percentage of correct classifications with respect to the total number of classified participants, as shown in Equation (2).

(2) $$Precision=\frac{TP}{TP+FP}$$

Recall is the percentage of correct classifications to the sum of correctly classified and incorrectly classified participants, defined in Equation (3).

(3) $$Recall=\frac{TP}{TP+FN}$$

F1 score provides the harmonic average between precision and recall as shown in Equation (4).

(4) $$F1=\frac{2\times \left(Precision\times Recall\right)}{Precision+Recall}$$

## 4. Results and Discussion

### 4.1. t-SNE Based Projection

**Figure 5:**
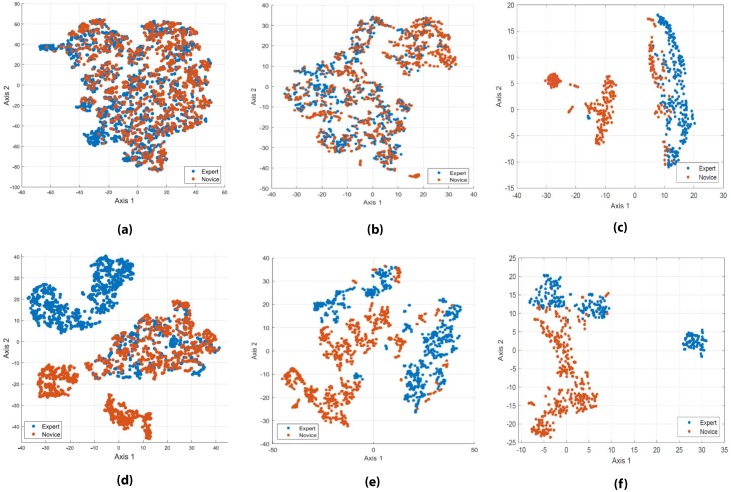
t-Distributed Stochastic Neighbor Embedding (t-SNE) of (**a**) ECG features without baseline correction; (**b**) GSR features without baseline correction; (**c**) Multimodal features without baseline correction; (**d**) ECG features with baseline correction; (**e**) GSR features with baseline correction; (**f**) Multimodal features with baseline correction.

The results of applying *t*-SNE to the features extracted from the ECG signals, both before and after baseline correction are shown in Figure 5a,d, respectively. The embedded feature space shows that the ECG features without baseline correction are not readily separable based on experts and novices. However, with baseline correction, the feature space appears to be separable based on participants level of expertise.

The feature space projection of the GSR features with and without baseline correction is shown in Figure 5b,e. Similar to the ECG *t*-SNE results, it can be seen that the features without baseline correction are not separable, while the features with baseline correction show better separability. However, there are still some overlaps between expert and novice groups. In our study only two levels of expertise were used: expert and novice. The presence of more granular representation of expertise between novice and expert (instead of a binary one) could account for this overlap and close clusters between some participants as some novices could be closer to expert level, while some expert participants may be closer to novice level. The overlap between the two groups could also stem from the differences in the participant’s basic ability to perform under different levels of cognitive load. An individual could innately have a greater or reduced physical response to cognitive stimulation when compared to other participants of their same class, novice or expert. This could allow for participants to be classified as having a different level of expertise.

*t*-SNE was performed on the multimodal feature set of ECG and GSR features with and without baseline correction, as shown in Figure 5c,f. Similarly with the individual feature sets, the features without baseline correction showed more overlap between classes, while the feature set using baseline corrected features shows clear separability. The results of combining the two feature sets show less overlap between classes than the *t*-SNE on ECG or GSR features individually. This finding suggests that a multimodal feature set using ECG and GSR signals is capable of differentiating expertise, supporting the development of the adaptive simulations based on the learner’s level of expertise classified from multimodal wearable data.

### 4.2. LASSO Feature Ranking

**Figure 6:**
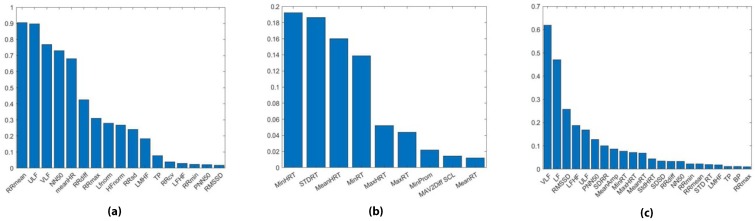
Regression coefficients for deterministic features using least absolute shrinkage and selection operator (LASSO) on: (**a**) ECG features; (**b**) GSR features; (**c**) multimodal (ECG and GSR) features.

As discussed in Section 3.2.4, the absolute values of the regression coefficients were calculated for each feature of the ECG, GSR, and multimodal feature sets. Using the values of the coefficients, the cutoff value for which features should be used within the classifiers was experimented with. It was found that using features with a regression coefficient above 0.01 achieved the best classifier results. The regression coefficients of those features are shown in Figure 6, where 17 features were found to have regression coefficients greater than 0.01. The most important features were found to be RR_mean_ and ULF with regression coefficients of 0.9044 and 0.8979, respectively. SDSD, LF, and HF were found to be the least important features (non-discriminative), with regression coefficients of 0. Generally the regression coefficients of the extracted ECG features tended to be high, indicating that ECG features are likely suitable for the classification of level of expertise.

From the regression coefficient of GSR features, shown in Figure 6, 9 GSR features were found to have regression coefficients greater than 0.01. The time domain GSR features were found to be more important than those from the frequency domain with the most important features being minimum HRT, standard deviation of RT, mean HRT, and minimum RT with regression coefficients of 0.1922, 0.1864, 0.1599, and 0.1387 respectively. None of the frequency domain features, or the features from SCR amplitude, or SCR area, were deemed discriminative as their regression coefficients were less than 0.01. There are fewer GSR features with regression coefficients greater than 0.01 when compared with the ECG feature set. Additionally the features that were found to be discriminative have lower values for their regression coefficients.

Our analysis on the LASSO regression coefficient of the multimodal feature set showed that 22 features had regression coefficients above 0.01. The ECG features had larger regression coefficients when compared to the GSR feature set. This is in line with our finding from using LASSO on the unimodal feature sets. The highest regression coefficient was from an ECG feature, namely VLF, with a value of 0.6245. In comparison, the highest regression coefficient for a GSR features was 0.0832, from mean SCR amplitude. More of the ECG features were found to be discriminative than GSR features. Our findings suggest that the ECG features are more important for differentiating between expert and novice classes for the purposes of future adaptive simulations.

### 4.3. Classification

Table 3 shows the results of using the classifiers to differentiate the level of expertise using LOSO validation scheme. The classifiers using the multimodal feature set performed better than the classifiers using either feature set separately with an accuracy and F1 score of 0.8296 and 0.7996 using the KNN classifier. These results differ from our findings based on LASSO as our earlier analysis showed that the ECG feature set was likely the most discriminative for level of expertise. It should be noted that while the results from LASSO can demonstrate which individual features are the most important for distinguishing between levels of expertise, the results do not indicate how well the features can be used together in a feature set for classification. Additionally, while LASSO used all of the participant data, LOSO validation was used during classification to set aside a subset of data for testing while the classifier was trained on the rest of the set. These differences could account for why the GSR and multimodal feature sets outperformed the ECG feature contrary to what the LASSO results suggested.

The high accuracy and F1 score from the LOSO classification results show the ability for the combined feature set to be used to differentiate between expert and novice classes, supporting our observations with *t*-SNE. These results demonstrate the ability of the classifiers to determine the level of expertise for new participants for which the system has not been trained. The accuracies achieved using LOSO show the feasibility of utilizing the proposed framework for dynamically adapting the simulation based on expertise. The multimodal feature set using both ECG and GSR will be used in further studies to develop more accurate and robust classifiers as it provided the best results when using LOSO validation.

## 5. Conclusions and Future Work

In order to classify expertise for an adaptive AR-based trauma simulation, we recorded ECG and GSR from expert and novice trauma responders. Feature extraction was performed in both time and frequency domains. The extracted features show a clear distinction between expert and novice trauma responders, with the combination of ECG and GSR features showing the most capability in differentiation. LASSO was utilized to identify the most important features for classification of expertise. Several classifiers were developed utilizing ECG, GSR, and multimodal feature sets that were found to be discriminative using LASSO regression. The KNN classifier, with *k* = 5, utilizing a multimodal feature set of 22 features outperformed the other models with an accuracy of 0.7852 and an F1 score of 0.7665. The findings show the feasibility of using ECG and GSR from wearable sensors for the classification of expertise. The results show promise that the classification can be integrated with our proposed system to allow for an increase in educational efficacy through the development of adaptive simulations tailored to the learner’s level of expertise.

For future work, the resolution of levels of expertise will be increased to include levels between expert and novice. This can be achieved through having an external reviewer grade the performance of each subject thereby allowing for a more accurate representation of the participant’s level of expertise. With an increase in the levels of expertise, simulations can be better fit to the learner and increase the efficacy of the learning objectives. Additionally, the differences between the signals recorded from experts and novices when dealing with the effects of adding visual complexity to the simulation through AR elements should be investigated. Future visual enhancements will be used to modulate the symptomology of the patient, to alter the difficulty of the simulation. The efficacy of these AR objects on successfully increasing cognitive load of the participant is crucial for the development of a dynamic simulation that can successfully increase the level of expertise of trauma responders. The optimal level of cognitive load that the participant should be put under to reach the desired learning objectives will also be investigated to successfully implement our proposed adaptive simulation framework. 

## Figures and Tables

**Table 1:** Time and frequency domain ECG features.

Feature	Description
RR_min_	Minimum value of RR interval
RR_max_	Maximum value of RR interval
RR_diff_	Difference between RR_max_ and RR_min_
RR_mean_	Mean value of RR interval
RR_SD_	Standard deviation of RR interval
RR_CV_	Coefficient of Variation of RR intervals
SDSD	Standard deviation of successive differences of RR intervals
NN50	Number of RR intervals greater than 50 ms
PNN50	Percentage of RR intervals greater than 50 ms
ULF	Ultra low frequency band (<0.003) Hz
VLF	Very low frequency band (0.04–0.003) Hz
LF	Low frequency band (0.04–0.15) Hz
HF	High frequency band (0.15–0.4) Hz
TP	Total power (0–0.4) Hz
LF_norm_	Normalized low frequency
HF_norm_	Normalized high frequency
LF/HF	Ratio of low to high frequency power
LMHF	Sympatho vagal balance ratio, (LF+MF)/HF, using mid frequency (MF) range of (0.08–0.15) Hz

**Table 2:** Time and frequency domain GSR features.

Feature	Description
RT	Rise time from SCR onset to peak response
HRT	Half recovery time of the SCR peak
Amp	Amplitude of the skin conductance response at its peak
Area	Area of the skin conductance response
Prom	Prominence of skin conductance response relative to the skin conductance level
SCL	Skin conductance level, the average electrodermal response
MAV1Diff SCL	First derivative of the mean absolute value of the skin conductance level
MAV2Diff SCL	Second derivative of the mean absolute value of the skin conductance level
BP	Band power power of the GSR signal
PSD	Power spectrum density estimate of the GSR signal

**Table 3:** Classification results using different feature sets with leave-one-subject-out validation scheme.

	SVM		DT		RF		KNN	
	Acc.	F1 Score	Acc.	F1 Score	Acc.	F1 Score	Acc.	F1 Score
ECG	0.7278	0.7398	0.6332	0.6454	0.7236	0.7270	0.5332	0.5234
GSR	0.7746	0.7712	0.7362	0.7123	0.7852	0.7665	0.7935	0.7889
ECG+GSR	0.7984	0.7815	0.7804	0.7931	0.6666	0.6804	0.8296	0.7996
